# Exploring barriers to assessment of bereavement risk in palliative care: perspectives of key stakeholders

**DOI:** 10.1186/s12904-015-0046-7

**Published:** 2015-10-14

**Authors:** Margaret Sealey, Moira O’Connor, Samar M. Aoun, Lauren J. Breen

**Affiliations:** School of Psychology and Speech Pathology, Faculty of Health Sciences, Curtin University, GPO Box U1987, Perth, WA 6845 Australia; School of Nursing, Midwifery and Paramedicine, Faculty of Health Sciences, Curtin University, GPO Box U1987, Perth, WA 6845 Australia

**Keywords:** Grief, Bereavement, Bereavement risk assessment, Palliative care, Family caregivers

## Abstract

**Background:**

Palliative care standards advocate support for grieving caregivers, given that some bereaved people fail to integrate their loss, experience ongoing emotional suffering and adverse health outcomes. Research shows that bereavement support tends to be delivered on an ad hoc basis without formal assessment of risk or need. To align support with need, assessment of bereavement risk is necessary. The overall aim is to develop a bereavement risk assessment model, based on a three-tiered public health model, congruent with palliative care bereavement standards for use in palliative care in Western Australia. The specific aim of this phase of the study was to explore the perspectives of key stakeholders and to highlight issues in relation to the practice of bereavement risk assessment in palliative care.

**Methods:**

Action research, a cyclical process that involves working collaboratively with stakeholders, was considered as the best method to effect feasible change in practice. The nine participants were multidisciplinary health professionals from five palliative care services, and a bereaved former caregiver. Data were obtained from participants via three 90 min group meetings conducted over five weeks. An inductive thematic analysis approach was used to analyse data following each meeting until saturation was reached, and the research team was satisfied that the themes were congruent with research aims.

**Results:**

Existing measures were found unsuitable to assess bereavement risk in palliative care. Assessment following the patient’s death presented substantial barriers, directing assessment to the pre-death period. Four themes were identified relating to issues in need of consideration to develop a risk assessment model. These were systems of care, encompassing logistics of contact with caregivers; gatekeeping; conflation between caregiver stress, burden and grief; and a way forward.

**Conclusions:**

These group discussions provide a data-driven explanation of the issues affecting bereavement risk assessment in palliative care settings. A number of barriers will need to be overcome before assessment can become routine practice. We recommend the development of a brief, pre-death caregiver self-report measure of bereavement risk that may empower caregivers, lead to early intervention, and allow staff to remain focused on patient care, reducing burden on staff and palliative care services.

## Background

The loss of a loved one is a normal, but nonetheless emotionally painful, life event that the majority of people integrate into their lives [1]. However, a minority of people, approximately 10 % of bereaved individuals, find adjustment difficult [2, 3]. Integration of the loss tends to be very difficult for these people and they may experience ongoing emotional suffering [4] which interrupts social, occupational, and physical functioning [5, 6]. This minority of individuals are more likely to be at risk of poor bereavement related outcomes such as poorer mental and physical health, and diminished quality of life [7, 8]. There is evidence to suggest that this minority of individuals suffer higher rates of cancers and cardio-vascular problems [9] as well as being at greater risk for suicide [10]. These complicated or prolonged grief reactions are supported by empirical research and criteria have recently been proposed for diagnostic nosology [11, 12]. Discussion on descriptive terms for this syndrome of reactions to grief has been contentious [13, 14]. Some argue that the term complicated may convey difficulty, whereas prolonged grief may provide clinicians with greater clarity, although Prigerson, Vanderwerker, and Maciejewski [3] caution against duration as the principle criterion. Despite such debates, few clinicians or researchers would contest that there are a minority of individuals adversely affected by grief and who require support to ameliorate such suffering [15]. In view of such concerns the *Diagnostic and Statistical Manual of Mental Disorders* (DSM-5) has categorised Persistent Complex Bereavement Disorder as requiring further investigation with a 12 month post-death period required before potentially meeting criteria for treatment [16].

Unlike many health settings, palliative care includes family caregivers within the unit of care [17] and, as such, offers a unique opportunity to assess grief and bereavement [18]. Palliative care standards and policies promote the provision of support to grieving caregivers where necessary [19]. Bereavement standards for specialist palliative care services [20] suggest assessing caregivers throughout pre-death contact with the service, using a structured assessment process and screening tool. As soon as possible following the death, caregiver distress due to the patient’s condition in the lead-up to death should be assessed, and within 12 weeks post-death all caregivers should receive follow-up telephone calls. The standards also recommend that at approximately six months after the death, those previously identified as ‘at risk’ should undergo further assessment with a validated measure such as the PG-13 (Prolonged Grief -13) [15]. However, research shows that bereavement support tends to be delivered on a piecemeal, ad hoc basis without formal assessment of risk or need [21–23]. This often results in support being offered to those who may not need it while others who would benefit are overlooked [24, 25]. This practice of blanket bereavement support contradicts findings showing grief intervention may be ineffective, or even harmful, for the majority who manage to integrate the loss into their lives [2, 26].

A three-tiered public health model of bereavement support articulated by Aoun, Breen, O’Connor, Rumbold, and Nordstrom [27] aligns intervention with need and is congruent with bereavement standards and policies. This model incorporates a low risk group of bereaved people that are likely to adjust in time with the support of family and friends; a medium risk group that would benefit from a volunteer-led, or peer support group, to prevent the development of ongoing issues; and a high risk group that would most likely require formal support from health professionals. Empirical support for this model has recently been demonstrated in a population-based survey [2].

To provide appropriate support in accordance with this model, bereavement risk assessment is the logical first step. Bereavement risk assessment in end-of-life care has been identified as a key indicator of quality practice [28]; however, at present, the majority of palliative care services do not use systematic, evidence-based methods to assess caregiver distress including bereavement risk [29]. Assessment is often centred on multidisciplinary team opinion and staff observation using in-house checklists [18, 30, 31]. An effective and reliable model of assessment is necessary to move towards best practice. The need for such a model has been supported internationally by palliative care services in 12 countries [32], as well as by local palliative care service providers, who identified bereavement risk assessment as a high priority [30] which provided the catalyst for this project.

The overall aim was to develop a bereavement risk assessment model for palliative care that would be congruent with standards and policies [20] and that would also be feasible for use in palliative care in Western Australia. As a first step in achieving this aim, we worked in consultation with a reference group comprising members from key Western Australian palliative care stakeholders via a series of group interviews. Where appropriate, the model would incorporate the use of existing grief measures, which were identified in a scoping review of the literature [33]. These measures were presented to the reference group for consideration as to their applicability for palliative care as part of the intended model. The specific aim of this phase of the overall study reported here was to explore the perspectives of key stakeholders in the reference group and to highlight issues in relation to the practice of bereavement risk assessment in palliative care.

## Methods

Data from the stakeholder discussions reported in this paper constitute part of one cycle within an overarching action research study. A key strength of action research is its capacity to tap local knowledge in research problems that relate to context-specific practices [34]. Action research is aimed towards intervention to bring about improvement in practice in a cyclic process of reflection, action and evaluation by the participants in the research activity [35–37]. While there are various definitions of action research the phase reported in this paper accords with Hart and Bond’s [35] professionalising type of action research where the research problem emerges from professional practice, is defined by the professional group, and resolution of the problem leads to enhanced professionalization of the service.

A characteristic and strength of action research is that the stakeholder participants have an active role in decision-making, while the investigator, rather than holding expert knowledge, takes the role of facilitating communication between group members [38]. The first author worked in palliative care clinical practice in the past, and as such, holds an insider perspective [39], which can be helpful on issues relating to clinical practice. However, there is also distance given the stakeholder group members were not known professionally to the first author. A reflexive journal was maintained in order to question assumptions and values and to generate critical enquiry.

Data were obtained from ongoing discussions with, and interaction between, the key stakeholders which enabled a range of perspectives, a shared understanding [40], and obstacles and potential solutions to emerge in the dialogue [41].

### Participants

Health professionals were recruited from five palliative care sites. The services were from government and non-government sectors. They included an in-patient palliative care unit, a consultative service in a major teaching hospital, a psycho-oncology service and two community-based domiciliary services. The reference group comprised nine members: two clinical nurses; a palliative care physician; a social worker; a psychologist; a counsellor; a psychosocial services manager; a chaplain; and a bereaved former caregiver who also worked as a palliative care volunteer. Ages ranged between 25 and 67 years (Mean 49.8), and years of experience ranged from less than a year to 25 years (Median 9 years). Most participants knew each other professionally.

### Procedures

The first author met with the managers of palliative care services in Perth, Western Australia in 2013 to capture operational information about the services. Requests for expressions of interest were sent to management at eight palliative care services, outlining what was required for participation in the project. Stratified sampling was used to select interested participants to represent the range of job designations across the various services and resulted in a group of health professionals typical of a palliative care multidisciplinary team, representing the breadth of models of care and services.

Once the reference group of key stakeholders was formed, three meetings were held in a centrally-located metropolitan health service meeting room. The second meeting was two weeks after the first, and the third meeting followed three weeks after the second meeting. The first author facilitated the meetings and digitally recorded the discussions, which were transcribed following each meeting. All meetings lasted 90 min.

The first meeting began with a discussion of the terms of reference for the group meetings, a brief background to the research question and the research objectives. The group was then asked for feedback on what they believed were necessary attributes of a bereavement risk assessment model for palliative care.

In the second meeting, existing self-report grief measures identified in a scoping review of the literature [33], were presented to the group and discussed. Seven measures from the scoping review were excluded: two were staff observational check-lists; one was a precursor to a later measure; one was a shortened version of a longer measure unavailable at that time; another related to suicide bereavement; one had yes/no responses to normal grief items; and one was a lengthy measure of normal grief. The 12 remaining measures were potentially suitable for use in palliative care at one of three time points; for use before the patient’s death (*n* = 3); in the month following the patient’s death (*n* = 5); and for assessing complicated grief at 6 months or beyond (*n* = 4). These times were in accordance with bereavement standards recommendations [20, 28]. Copies of the measures, and a summary of research articles pertaining to the measures, were given to participants who agreed to return to their services and discuss the materials with their multidisciplinary teams.

The third meeting was used to explore in detail each of the measures presented at the previous meeting, and to examine their suitability for a bereavement risk assessment model.

### Ethics approvals

In compliance with the Helsinki Declaration, Human Research Ethics Committee (HREC) approvals were obtained prior to commencement from two major teaching hospitals and the university:-Royal Perth Hospital (approval number EC2012/167), South Metropolitan Health Service (approval number R/13/17) and Curtin University (approval number HR30/2013). All participants gave written consent to participate in the research.

### Analysis of data

Data were analysed using an inductive thematic analysis approach in accordance with Braun and Clarke’s [42] six phases. Each digital audio recording was repeatedly listened to by the first author to maximise familiarity with the semantic content. The recordings were transcribed verbatim by the first author as soon as possible following each of the meetings. Each transcript was manually coded and preliminary themes were developed prior to the next meeting, allowing the first author to commence each meeting with a summary of interim findings which was confirmed by participants. The feedback from the participants during these meetings, and in telephone and email correspondence between them and the first author, were also used as data. Further scrutiny of transcripts and interim analyses by the research team revealed that saturation had been reached with sufficient information from the participants to illustrate the issues across the different services [43]. The themes have been illustrated below using the participant’s own words, thus retaining participants’ viewpoints.

## Results

Thematic analysis of the data revealed four themes in relation to potential use of existing measures. These were systems of care, encompassing logistics in contacting caregivers; gatekeeping; conflation between caregiver stress, burden and grief; and a way forward. The first three themes are considered in the context of pre and post-death assessment concluding with a way forward.

### Pre-death assessment of grief

The pre-death period was regarded as providing the best opportunity for assessment because this is when most services have face to face contact with caregivers. Three pre-death measures were considered by the reference group in detail. The Marwit-Meuser Caregiver Grief Inventory (MM-CGI) [44] received the greatest support, with the short form version [45] deemed unable to capture sufficient information. However, because the MM-CGI was developed for family caregivers of people with dementia, the items would require considerable re-working and subsequent validation before being of potential use in palliative care. The Clinical Psychologist noted that re-wording items on the measure “… *might affect the factor structure of the questionnaire.”* She gave an example of an item on the MM-CGI relating to the closeness of a loved one, stating: *“…cognitive decline and connectedness with my family”* may be a different experience *“through the experience of cancer and caring for someone* [when] *it actually increases the connectedness.”*

In considering Prigerson and colleagues’ PG-12 caregiver measure [46], participants found an item relating to “moving on” unhelpful, and believed this measure could confuse caregivers. The Bereaved Former Caregiver said she “*would be put off if I was asked to rate that.”* The Clinical Nurse Manager from the community service said that their service would not use it, stating: “*it’s too early to expect someone to have moved on. To be honest, if I was a carer, I think I’d be offended by that.”*

While the pre-death period seemed optimal for the assessment of caregiver bereavement needs, discussion centred on the many issues affecting bereavement assessment in palliative care. Themes emerged in relation to challenges stemming from systems issues, such as differences in service models which affect patient contact and the logistics of assessment when a patient is near death. Strongly woven into these issues was a tendency toward staff gatekeeping in order to shield family caregivers from emotionally loaded situations thus adding to their burden. Conflation between grief and caregiver stress and burden also emerged as a salient theme.

### Systems of care

Systems issues related to each service’s model of care and funding source which influenced staffing, length of patient stay in a service, and type of support they could provide. By necessity patients often move between the different types of services where they are *“…going to probably be connected with a range of different services, because of the interdisciplinary approach that a specialist palliative care service”* uses (Support Services Manager, community service). The Physician from the in-patient unit stated: *“…people move around the system, contacting community services, hospice services and we try to be aware of that.”*

Because patients use multiple services there is often no clear delineation as to which service is responsible for bereavement care. The Physician added that when people may “…*need access to bereavement services… there’s a case for where the responsibility should lie, or if there’s duplication of services…there can be a predominant service they* [patient and family] *have been involved with. That’s the most appropriate thing for them, but I think it’s awfully ad hoc”.* Compounding this, without medical records available across services, information as to whether support has been accessed by family caregivers is rarely available.

As patients move around the system and between multiple services, there is a potential for them, and caregivers, to be missed between services. As the Bereaved Former Caregiver said: *“I do believe that ‘slipping through the system’* [being missed between services] *unfortunately happens a lot. I wasn’t in a system where anybody phoned me. I as the carer needed help. I didn’t know where to go.”*

Where patients are in the care trajectory influences what model of care they receive. For example, a community domiciliary service generally has referrals earlier in the palliative care trajectory, whereas a consultative service in a major hospital may only receive referrals when all other treatment options have been exhausted and the patient is near end-of-life. Contact with patients, and consequently their caregivers, would strongly influence the logistics of when, or if, a measure would be administered:

*“When I think about the sort of situations we have and the acuity and intensity, rapid changes, and multiple people involved with family and complicated circumstances, some people are absolutely in no shape to engage in any of this sort of discussion actually.”* (Physician, in-patient unit)

Further challenge to assessment relates to the provision of appropriate support services, either within the palliative care service itself, or as part of a referral pathway to external services. Some reference group members questioned the use of measures if the relevant support was unavailable:

*“*[An assessment tool] *needs to be seen within a service context, because to just apply it without any context whatsoever, you are then up against this issue of, well, how valid is it going to be?”* (Psychosocial Support Services Manager, community service)

### Gatekeeping

Gatekeeping, as a process of deciding on the allocation of services [47], was evident in the participants’ reticence to engage caregivers directly in relation to their emotional needs. As noted by the Clinical Nurse Manager from the community service:

*“We’ve all discussed that we wouldn’t do the assessment as we do the admission process, so it was waiting ‘til people go into that deteriorating phase, but not into the terminal phase, because we want to sort of gauge that… you’re a little hesitant to actually pick that time… It would just be overwhelming.”*

Concern was expressed about being sensitive towards caregivers particularly in relation to asking personal questions. As the Physician said in relation to the use of a clinical assessment tool, it “…*is a very sectioned process, and working out private information for people.”*

However, the Bereaved Former Caregiver, when questioned as to whether she would have felt overwhelmed by ‘insensitive’ questions, indicated that caregivers have a choice as to whether or not they engage, stating that *“I personally wouldn’t have taken offence…I guess if you’re not willing to do it, you just ignore it. And I think it comes with when you’re ready.”*

The Psychologist suggested that asking another family member, or friend, to assess how they believed the caregiver was coping would be beneficial; however, others believed this would be unworkable. The Physician responded, stating: *“I need to think a bit about that component of the assessment which may require a carer to conform…which is again why I say our team assessment is very much a case of getting a sense of the whole situation. Sometimes the primary carer seems to change over the course of time they are with us.”*

Alongside the desire to protect caregivers from further distress there was a preference for an informal chat rather than engaging the caregiver in a direct and formal discussion about how they’re coping. As the Clinical Nurse Manager from the community service pointed out: “…*it’s going to be more of a conversation that you have as you go to the car as to whether they would be part and parcel of this* [self-report assessment]*.”*

The participants believed that it was preferable to gauge the family caregiver’s journey through observation and by looking at where they were in the palliative care trajectory, rather than using a formal measure by “…*having a look at how the person moves through the process. It’s not always going to be possible to apply a tool. It’s going to be based upon observation* [and] *interaction”* as noted by the Psychosocial Support Services Manager from a community service. He added that by the time a patient reaches end-of-life, the multidisciplinary team has “…*been able to come to a view… on the basis of a picture that’s emerged over a period of time, that can help influence whether you know that person is identified as being at low, moderate, or high risk.”*

Observation, informal chat, intuition, and guesswork were prioritised over formal assessment of the caregiver:

*“I guess part of our job in hospice care is to walk along with them* [patients and families] *and some of those things are appropriate, and some of them do pass because the experience changes them. And that’s why those interactions and discussions that come from that place are very important, because you know people can say ‘I had this conversation last night and I sense it was an issue’.”* (Physician, in-patient unit)

The participants valued the professional judgement that is at the core of their multidisciplinary team’s assessment. However, there were flaws in checklists and as noted by the Physician: *“…the information is usually left and filled-out at the time of the death, when it should have been filled-out sooner.”*

In spite of problems relating to the accuracy of information on the checklists filled-in by staff regarding their observations of caregivers, they were still thought valuable in building an overall picture:

*“Our team assessment is very much a case of getting a sense of the whole situation, who the different people are…the tick boxes were just to give the team a reminder to attend to things that they can head off along the way…for us it’s more about a team sense of what the issues will be. It is just purely reminding us of issues as we come across, that as a team we sort of say ‘what do we think? How can we follow up?’”* (Physician, in-patient unit)

#### Conflation between caregiver stress, burden and grief

Caregiving is a time of potential high distress given the likelihood of caregiver burden during the patient’s illness [48] where distress might cloud assessment. The Counsellor from a community service pointed out:

*“…there are significant milestones for people in grief…three months and sometimes 12 months can be a significant time when you can catch a person in a bad week”.* The Social Worker from the consultancy service agreed stating: *“when I do a follow-up in a year, you actually get more people coming back saying ‘I’m not doing okay.’ It could be because it is around that year anniversary.”*

Further complexity may be added to assessment by other factors:

*“The person who is generally caring for the person who is dying, is probably caring for a lot of other people…it’s generally the mother, but not always. One of the aspects I come across quite a lot is that when it comes to assessing how people are grieving as a family, there’s never two people on exactly the same page, and then that becomes complex. So I guess, the person about who’s being assessed, is about who they are supporting as well.”* (Counsellor, community service)

A further complicating issue was the need to assess trauma as well as grief at times. This would not be required as routine assessment but would be helpful in screening for the likelihood of posttraumatic stress disorder (PTSD) so that timely referral and intervention might avoid later complex issues:

“*At our meeting today we were discussing someone who had died during the week who had one of those catastrophic bleeds. The son was the one who discovered it. There’s trauma as well as grief in the event of the death.”* (Counsellor, community service)

This conflating of grief with other psychological issues reflects a lack of clarity about the purpose of assessment, and a tendency to focus on immediate needs related to caregiver stress in order to provide a solution:

*“We’re also talking about what services need to do in order to discharge their obligations to people. Who do they contact to make sure they identify people who may need extra help?”* (Physician, in-patient unit)

*“I guess for me it’s a question too of what are we assessing for? Are we assessing to identify people who are at risk of PGD* [Prolonged Grief Disorder]*? Or are we assessing what are the issues that are confronting particular people that will allow a service to say, ‘these are the things that this person might need to deal with what has just occurred in their life?’”*(Counsellor, community service)

### Post-death assessment of grief

Asking participants to choose measures for use in the post-death period proved more challenging than at pre-death, largely because services have little or no face-to-face contact with family caregivers. For the immediate post-death period, five measures were considered; the Core Bereavement Items (CBI) [49]; the Grief Evaluation Measure (GEM) [50]; the Hogan Grief Reaction Checklist (HGRC) [51]; the Two-Track Bereavement Questionnaire (TTBQ) [52]; and the Texas Revised Inventory of Grief (TRIG) [53]. Of these, the GEM’s ‘experiences’ section, and the TTBQ were both considered as yielding the most comprehensive and clinically useful information; however, both were deemed overly long and complex for telephone administration. The shorter CBI and TRIG measures were considered as alternatives, but concerns were raised in relation to their potential to screen for prolonged or complex grief issues after the death. The HGRC likewise was regarded as picking up on ‘normal’ grief, but was thought to have an added disadvantage of 61 items, which would be particularly unwieldy via telephone.

Measures proposed for PGD at six months following a patient’s death were the Inventory of Complicated Grief (ICG) and its revised version (ICG-R) [54]; the Prolonged Grief – 13 (PG-13) [12]; and the Brief Grief Questionnaire (BGQ) [55]. The services do not assess bereaved former caregivers for PGD or Complicated Grief (CG) at six months, and as such, the reference group health professionals had difficulty considering the prospect of doing so due to present systems issues, especially funding constraints. As the Clinical Nurse Manager said: *“I didn’t have any huge feelings about any of them* [measures for CG or PGD] *to be honest with you.”* The community service she represented kept caregivers *“…on the books for 4 to 6 months, and if one of the staff feels that they need to stay longer than that, then so be it.”* The Clinical Nurse Manager from the consultancy service stated: *“We just don’t have contact with carers at this time. We don’t have the resources to call. This* [a measure for prolonged or complicate grief at six months] *isn’t something we would use.”*

### Systems of care

There was agreement that an issue for services was the length of time bereavement support could be offered to people for whom they did not receive funding:

*“Certainly it is for us, and I think it’s an issue for a lot of specialist palliative care services, the very nature of how we’re structured, the bereavement support we can provide is very limited, and often bereavement support is very closely identified with specialist palliative care, when very clearly, they’re actually separate. They’re related in relation to a service, but they’re* [bereavement support and palliative care service] *quite separate because we really need to be concluding our involvement within a three to four month period. Clearly that’s going to be missing out on a whole lot of potential issues that could arise.”* (Psychosocial Support Services Manager, community service)

As none of the services had a dedicated bereavement staff member, follow-up fell to various team members as an additional role. Chaplains conducted the bereavement care at the in-patient unit, and it was the role of the social worker at the consultancy service. Counsellors at one community service (the only service with a counselling team), tracked bereavement care with people who had been identified as needing follow-up using the Bereavement Risk Index (BRI), a staff-completed observational checklist [56]. At the other community service, bereavement follow-up was conducted predominantly by nursing staff. Follow-up at other services was based on telephone calls and individual circumstances. The lack of a dedicated staff member to take responsibility for bereavement care contributed to inconsistency in follow-up contact:

*“…so up ‘til now the BRI* [Bereavement Risk Index] *process has essentially been nurse led. Something that we’re just pushing through at the moment is that where a counsellor is involved with the person pre-death, it’s the counsellor’s job to drive the bereavement process.”* (Psychosocial Support Services Manager, community service)

*“You mark it in the diary that it is designated to all the team, you know ‘this bereavement call is due on this day’.”* (Clinical Nurse Manager, community service)

The length of time spent in maintaining these tenuous links with bereaved former caregivers also varied between services*“…from three or four calls”* as indicated by the Chaplain from the inpatient unit to 12 months post-death at the consultancy service. The Psychosocial Support Service Manager from the community service said: *“We really need to be concluding our involvement within a sort of 3 to 4 month period.”*

The focus of palliative care is on patient care in the pre-death period, which poses challenges for staff’s support of family caregivers:

“…*particularly where you know the service has basically withdrawn to a large extent. This is probably the last, if the only remnant of the services from* [name of service]…*it’s a full blown service while the person is alive and being cared for in palliative care, but now it’s just down to one person contacting over the ‘phone. So it’s diminished in that regard.”* (Counsellor, community service)

Typically, follow-up by all the services included telephone calls, a remembrance card at 12 months, and an invitation to a memorial service, rather than assessment of support needs:

*“We send a card, then we have a service. The most contact we have from carers is from our annual remembrance card and it’s amazing the number of times we get a phone call back after that, or a card to say ‘we can’t believe you actually remembered’ the person.”* (Clinical Nurse Manager, community service)

### Logistics of maintaining contact with former caregivers

Most services do not have face-to-face contact with bereaved caregivers following the patient’s death which raised a number of problems. A measure comprising a number of items, with a variety of responses, would be very difficult to complete by telephone. While all participants agreed that a more structured assessment would be helpful, a self-report measure would not be feasible in the weeks or months following a death:

*“I looked at all of them* [grief self-report measures] *and I found them all very difficult to be able to use over the ‘phone. I guess the only thing for us* [government community service] *it would perhaps mean we would need to re-think the way we do our bereavement* [support]*, and for the first month instead of ‘phone contact make a visit to make it a workable thing.* (Clinical Nurse Manager, community service)

Bereaved former caregivers could be difficult to contact after the patient’s death:

*“Some of those demographic details, and conditions of people, their lives may have changed. They may have changed address for example.”* (Counsellor, community service)

Follow-up contact was also time-consuming and often overlooked when bereaved former caregivers were not answering calls:

*“You make a ‘phone call, you can’t get through. The following day you make a ‘phone call, but unfortunately the busy-ness of people that are still with us, then those ‘phone calls get lost along the way, and beneath the week, you mark it in the diary that it is designated to all the team, you know ‘this bereavement call is due on this day’. If you’ve done it perhaps three times, we don’t then follow it up. If you’ve missed somebody three times, we go ‘okay we’ll go to the next month’, so there is actually no way of finding out perhaps if a carer is okay, or not okay. It just gets lost I think in the busy-ness of how we are.”* (Clinical Nurse Manager, community service)

### Gatekeeping

Palliative care teams are reluctant to refer caregivers on, preferring to support them within the team:

*“I think it’s very natural in palliative care teams to follow-up, well initially, but that sense of being able to hand over to a formal structured bereavement service…for people falling outside the normal is, I think, something that we probably in palliative care have been guilty of retaining ownership of…it’s very hard for us to not let go of the fact that we should be doing something…there are times we shouldn’t be doing anything.”* (Physician, in-patient unit)

While all services provided some follow-up of bereaved former caregivers identified as being at potential risk of poor bereavement outcomes at multidisciplinary team meetings, this was often based on intuition as to whether follow-up should proceed. As the Clinical Nurse Manager from the community service said: “…*it’s very much a gut feeling, or when you make a phone call to that person, how they respond I guess.”*

### The way forward

Because existing instruments were found unsuitable for use in an assessment model, the Physician from the inpatient unit stated: *“Can I recommend that you make a new one* [measure]*?”* The participants suggested that a measure be constructed that could be tailored for use in palliative care, prior to the patient’s death in order to assess the caregiver’s bereavement risk.

A new measure should be brief and easy for caregivers to use, simple for staff processing and documentation, and would ideally account for the known risk factors for poor bereavement outcomes. As the Clinical Nurse Manager from the community service said, *“bereavement stuff, for it to be consistently used we need something simple. If it’s a big piece of paper we’re not going to do it. We’re going to put it down to the bottom of the pile.”* The Clinical Psychologist suggested focusing on *“…the main risk factors for bereavement. Whether its previous mental health sort of diagnosis, where there are low levels of social support, low economic, you know, low SES* [socioeconomic status]*, all those sorts of things.”*

## Discussion

While the different disciplines and service models of care varied between the reference group members, their data formed a cohesive explanation of the barriers to bereavement risk assessment in palliative care [43]. None of the 12 self-report measures was considered to be suitable for use in palliative care; as such, the initial aim to select existing grief measures for use in a bereavement risk assessment model was not achieved. When considering bereavement risk assessment, the participants highlighted several barriers to the use of existing measures in their services. Specifically, they described the ways in which systems of care, logistics in relation to contact with former caregivers, and conflation between caregiver stress, burden and grief affects the ability to assess caregiver grief. The specific barriers changed between the pre-death and post-death period. The issues in the pre-death period largely centred on staff’s reluctance to ask intrusive or sensitive questions as end-of-life approached. In the post-death period, contact with caregivers was difficult due to various barriers such as staffing, funding, and availability of contact with caregivers.

### Pre-death assessment

In spite of the many challenges to assessment before the patient’s death, this period presented the best opportunity for services to assess caregiver bereavement risk because caregivers have face-to-face contact with staff. As in findings by Agnew et al. [18], the health professional participants described flaws in the current use of staff-completed checklists where the checklists are generally completed following the patient’s death with information that is often based on staff intuition. Therefore, the participants highlighted the need to develop a new measure that would include known risk factors for complicated or prolonged grief, yet be brief and user-friendly for both caregivers and palliative care service use. If a more robust assessment of caregiver bereavement risk could be achieved at this time caregivers could be provided with assistance tailored to their needs [27]. This project has identified the hurdles to be overcome, so that this aim can be achieved.

Palliative care service models will no doubt continue to vary due to the nature of the service and their funding. As such, patients (and their caregivers) will continue to move between services within the system. A standardised bereavement risk assessment protocol, transferrable across services, would be beneficial for both caregivers and staff in order to identify caregiver bereavement needs in a timely manner, thus minimising duplication in assessment and the provision of support, and reduce the likelihood of caregivers being missed between the various services in the system. While staff focus and palliative care resources remain primarily on patient care in the lead-up to the patient’s death [57], the development and use of a validated brief caregiver self-report measure could enable staff to continue patient care with more assurance that family caregivers’ emotional needs are being met, whilst ensuring that caregivers’ issues are not a distraction from patient focused care. Standardised assessment across the palliative care trajectory should also have the added benefit of tracking change for caregivers across time, and more readily identify which of the available palliative care services might be best placed to provide support.

The introduction of a new measure may also alleviate the problems related to staff gatekeeping where decisions on the allocation of support are based on staff preference to intuit caregiver needs rather than asking caregivers to report their needs. The Carer Support Needs Assessment Tool (CSNAT), which asks caregivers to state their needs, has provided structure and guidance to previously undocumented, informal conversations [57]. In Australia [58] and the United Kingdom [59] caregivers identified that dealing with their feelings and worries was in the top three of their needs, indicating that the avoidance of directly assessing caregivers’ needs and distress is contrary to what caregivers themselves want. This formal identification of caregiver concerns led to early intervention, and resulted in positive outcomes for caregivers in reducing caregiver strain. Similarly a self-report measure of bereavement risk may allow caregivers to voice their feelings and worries without staff discomfort about having sensitive conversations.

The reference group health professionals stated that the multidisciplinary teams are well able to build a comprehensive picture of families’ needs during the palliative care trajectory. While this is a legitimate and valuable method of assessment, the addition of a caregiver self-report would move towards a caregiver-led [57], person-centred approach, rather than the present paternalistic model based on ‘expert’ judgement and observation of caregiver responses. It should also move away from the present practice of staff observation and discussion that is undertaken without caregiver knowledge or consent [21]. A caregiver self-report measure could empower caregivers and assist palliative care staff to intervene where appropriate. Assessment to identify those at risk of complicated or prolonged grief, and referral where required, needs to become standard practice to circumvent potential ongoing mental health issues [60, 6]. Boerner, Mancini, and Bonanno [61] advocate that health professionals are ideally placed to gather such information prior to a death and address the issues by referring those at elevated risk to clinicians or services who may provide appropriate support in a timely manner.

The reluctance to address caregiver emotional needs directly may stem from misunderstandings by health professionals about the grieving process. Powazki et al. [62] found that education for nurses relating to end-of-life care and communication was lacking. Surveys of university courses also reflect a similar lack of education that would prepare health professionals across disciplines to provide grief support [63]. General Practitioners also tend to try and resolve patients’ grief themselves using psychotherapeutic strategies without the specialist training required [64]. Such lack of understanding by health professionals in relation to grief may account for the belief by reference group participants that they should ‘do something’ for the majority who do not need anything other than the support of family and friends.

Findings support previous research suggesting that palliative care multidisciplinary team members often believe they are best placed to provide psychosocial support, and indicate that there is a reluctance to refer to appropriate support services [65]. A caregiver self-report measure of bereavement risk may clarify for staff when referral is needed, accompanied by a referral pathway to appropriate support. However, education will be needed to encourage staff to incorporate such assessment and sensitive conversations into routine practice. Education about all aspects of grief, particularly in relation to staff judgements and intuition about caregivers’ emotional responses, needs to occur across the gamut from university education to professional development programs [62].

### Post-death assessment

For the period following the patient’s death, both in the short term and longer term, the greatest challenges to bereavement risk assessment stem from logistical barriers in maintaining contact with former caregivers. The participants believed that existing self-report measures would be too long and complex to administer via telephone, given this was generally the only means of contacting caregivers. Even if services had staff trained in assessment and dedicated to bereavement care, the data from this study suggest the availability of appropriate bereavement services and referral pathways is lacking. This issue could be addressed by establishing referral pathways to appropriate community services [64]. As Rumbold and Aoun [66] suggest, palliative care services would do well to forge connections with community services that could meet the needs of this group so as to minimise the possibility of their developing ongoing health issues.

Likewise, the use of a prolonged grief measure at six months following the patient’s death, as per bereavement standards [20], also proved challenging. The DSM-5 states a 12 month period should elapse before bereavement related criteria can be met. However, for those with co-morbid depressive diagnoses, bereavement has been removed as an exclusion to treatment, allowing this group to receive help sooner rather than later [67]. Research indicates that caregivers are at higher risk of depressive symptomatology with many meeting clinically significant criteria in need of assistance [68, 69], adding weight to the argument for earlier, more robust assessment to identify those in need of follow-up to prevent ongoing health issues.

### Bridging policy and practice

Bereavement support standards for specialist palliative care services [20] have only recently been developed and, as such, have provided an initial, much needed framework to address the many complexities of bereavement care. The standards recommend that universal strategies such as screening and risk assessment, and supportive programs and information should be extended to all caregivers across the palliative care trajectory. Specialist bereavement support strategies including counselling and psychotherapy and/or bereavement support groups should be offered to those currently distressed or at elevated risk of complex grief related issues. This protocol is supported by the public health model [27] articulating three tiers of need.

In the United States of America, hospices are required by medical insurance funders to provide assessment of needs in relation to bereavement care for approximately 12 months following a patient’s death [70]. While this is not yet current practice in Australia it is becoming increasingly likely that it may become so. Accreditation for health services, including hospitals and palliative care services, is already linked to compliance with standards [71] with funding expected to be linked in the foreseeable future [72]. Should these conditions be required, palliative care services will need to address bereavement risk assessment as a matter of greater priority than has already been proposed [73].

### Strengths, limitations and future research

These stakeholder group discussions provide a data-driven explanation of the issues affecting bereavement risk assessment practice in a variety of palliative care service settings. The strength of this study was the composition of the reference group. There was a diversity of health professional designations typical of a multidisciplinary palliative care team, as well as a bereaved former caregiver. This diversity facilitated an opportunity for various perspectives on data collected in relation to the research question, which increases the study’s capacity to inform practice [43]. A greater number of people in the reference group, including more bereaved former caregivers, may have added diversity to the opinions in the group, but may also have decreased the opportunity for all members to contribute. Given the specific focus of the group, the number of participants was chosen to optimise the involvement of all participants [74, 75]. Nevertheless, the findings may not transfer to other locations with different palliative care service models although research shows that similar challenges to bereavement care exist in other developed countries [23]. Due to the many difficulties with post-death assessment highlighted by the reference group future research should focus on the development and testing of a pre-death measure of bereavement risk that is feasible for palliative care settings, particularly given the consequences this minority of individuals face in terms of poor health [7]. If a measure could assess for the risk factors in complications of bereavement and predictors of bereavement outcomes, then palliative care service providers could refer caregivers at elevated risk to appropriate health professionals or services for appropriate monitoring and support [61].

## Conclusion

The broad aim of this study was to develop a bereavement risk assessment model using existing measures that would be congruent with bereavement support standards [20]. The phase of the overall study reported in this paper examined the measures in collaboration with a reference group of palliative care stakeholders via a series of group interviews. However, existing measures were found to be unsuitable. A number of barriers will need to be overcome before assessment can become routine practice. The barriers are associated with system of care issues, such as multiple service use and availability of support personnel; and logistics relating to service contact with caregivers, where contact following the patient’s death is difficult due to funding and problems with telephone contact. Staff gatekeeping, where follow-up support is often determined through intuition, as well as conflation between caregiver stress, burden and grief were also identified as problematic in risk assessment.

We recommend the development of a brief caregiver self-report measure of bereavement risk to allow caregivers to voice their worries and concerns [59] allowing staff to remain focused on patient care. This may reduce burden on both staff and palliative care services. Comprehensive assessment of caregiver bereavement risk may more readily ascertain the type of support a bereaved former caregiver may need, thus allowing palliative care services to provide appropriate support or referral to other organisations specialising in bereavement support [66].

## Abbreviations

BGQ: Brief Grief Questionnaire
BRI: Bereavement Risk Index
CBI: Core Bereavement Items
CG: Complicated grief
CSNAT: Carer Support Needs Assessment Tool
DSM-5: Diagnostic and statistical manual of mental disorders-5 (5^th^ Edition)
GEM: Grief Evaluation Measure
HGRC: Hogan Grief Reaction Checklist
ICG: Inventory of Complicated Grief
ICG-R: Inventory of Complicated Grief-Revised
MM-CGI: Marwit-Meuser-Caregiver Grief Inventory
PG-12: Prolonged Grief-12
PG-13: Prolonged Grief-13
PGD: Prolonged Grief Disorder
PTSD: Posttraumatic Stress Disorder
SES: Socioeconomic status
TRIG: Texas Revised Inventory of Grief
TTBQ: Two-track Bereavement Questionnaire
